# State-of-the-art retinal vessel segmentation with minimalistic models

**DOI:** 10.1038/s41598-022-09675-y

**Published:** 2022-04-13

**Authors:** Adrian Galdran, André Anjos, José Dolz, Hadi Chakor, Hervé Lombaert, Ismail Ben Ayed

**Affiliations:** 1 University of Bournemouth, Bournemouth, UK; 2 Idiap Research Institute, Martigny, Switzerland; 3 ETS Montréal, Montreal, Canada; 4 Diagnos Inc., Quebec, Canada

**Keywords:** Biomedical engineering, Computer science, Machine learning

## Abstract

The segmentation of retinal vasculature from eye fundus images is a fundamental task in retinal image analysis. Over recent years, increasingly complex approaches based on sophisticated Convolutional Neural Network architectures have been pushing performance on well-established benchmark datasets. In this paper, we take a step back and analyze the real need of such complexity. We first compile and review the performance of 20 different techniques on some popular databases, and we demonstrate that a minimalistic version of a standard U-Net with several orders of magnitude less parameters, carefully trained and rigorously evaluated, closely approximates the performance of current best techniques. We then show that a cascaded extension (W-Net) reaches outstanding performance on several popular datasets, still using orders of magnitude less learnable weights than any previously published work. Furthermore, we provide the most comprehensive cross-dataset performance analysis to date, involving up to 10 different databases. Our analysis demonstrates that the retinal vessel segmentation is far from solved when considering test images that differ substantially from the training data, and that this task represents an ideal scenario for the exploration of domain adaptation techniques. In this context, we experiment with a simple self-labeling strategy that enables moderate enhancement of cross-dataset performance, indicating that there is still much room for improvement in this area. Finally, we test our approach on Artery/Vein and vessel segmentation from OCTA imaging problems, where we again achieve results well-aligned with the state-of-the-art, at a fraction of the model complexity available in recent literature. Code to reproduce the results in this paper is released.

## Introduction

**Figure 1:**
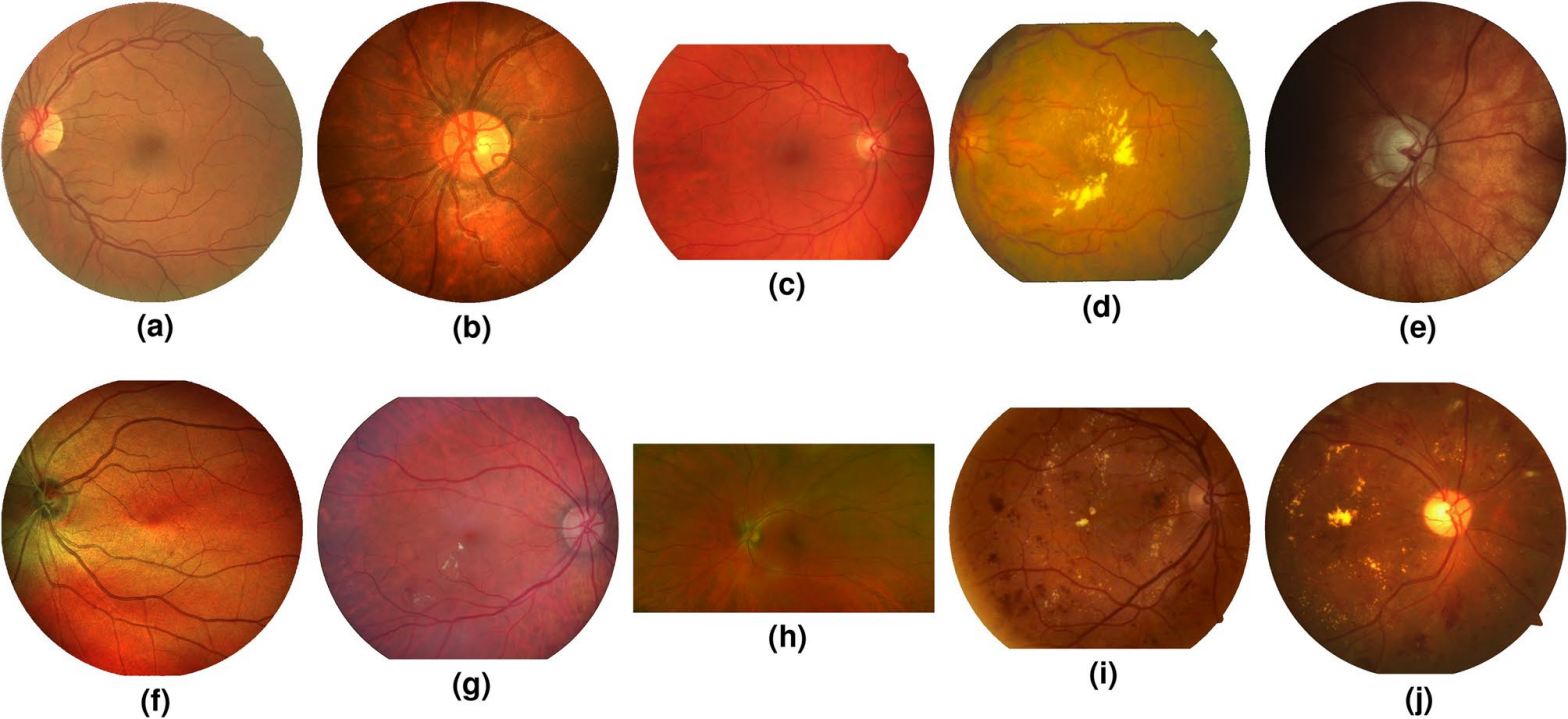
This work provides a comprehensive cross-dataset performance study on vessel segmentation. This figure shows a representative image from each of the 10 databases used in this paper: (**a**) DRIVE^1^, (**b**) CHASE-DB 1^2^, (**c**) HRF^3^, (**d**) STARE^4^, (**e**) LES-AV^5^, (**f**) IOSTAR^6^, (**g**) DR HAGIS^7^, (**h**) AV-WIDE^8^, (**i**) DRIDB^9^, (**j**) UoA-DR^10^. A detailed description of each database is given in Table 2.

Retinal vessel segmentation is one of the first and most important tasks for the computational analysis of eye fundus images. It represents a stepping stone for more advanced applications such as artery/vein ratio evaluation^11^, blood flow analysis^5^, image quality assessment^12^, retinal image registration^13^ and synthesis^14^.

Initial approaches to retinal vessel segmentation were fully unsupervised and relied on conventional image processing operations like mathematical morphology^15,16^ or adapted edge detection operations^17^. The idea behind these methods was to preprocess retinal images to emphasize vessel intensities. Preprocessed images were then thresholded to achieve segmentation. While research on advanced filtering techniques for retinal vessel segmentation continued over more recent years^6,18^, such techniques consistently fail to reach competitive performance levels on established benchmarks, likely due to their inability to handle images with pathological structures and generalize to different appearances and resolutions.

In contrast, early learning-based approaches quickly showed more promising results and better performance than conventional counterparts^1,19–22^. The common strategy of these techniques consists on the extraction of specifically designed local descriptors that are followed by a relatively simple vessel classifier. Contributions found in literature mostly focus on the development of new discriminative visual features rather than on the classification sub-task.

The predominance of Machine Learning (ML) techniques was reinforced with the emergence of deep neural networks. After initial realization that Convolutional Neural Networks (CNN) could outperform previous methods, bypassing any manual feature engineering, and directly learning from raw data^23,24^, a constant stream of publications has emerged on this topic, up to the point that almost any new competitive vessel segmentation technique is now based on this approach.

Standard CNN approaches to retinal vessel segmentation are based on the sequential application of a stack of convolutional layers that subsequently downsample and upsample input images to reach a probabilistic prediction of vessel locations. During training, weights of the network are iteratively updated to improve predictions by means of the minimization of a miss-classification loss (e.g. Cross-Entropy). Either processing small image patches^23^ or the entire image^24^, these approaches can succeed in segmenting the retinal vasculature only relying on a relatively small set of annotated samples.

Extensions to the CNN paradigm tend to involve complex operations, such as specifically designed network layers. Fu et al.^25^ introduced a Conditional Random Field recurrent layer to model global relationships between pixels. Shi et al.^26^ combined convolutional and graph-convolutional layers to better capture global vessel connectivity. Guo et al.^27^ introduced dense dilated layers that adjust the dilation rate based on vessel thickness, and Fan et al.^28^ proposed a multi-frequency convolutional layer (OctConv). Other custom convolutional blocks and layers based on domain knowledge were explored in recent works^29,30^.

Specialized losses were also proposed in recent years. Yan et al.^31^ trained a U-Net architecture^32^ by minimizing a joint-loss that receives output predictions from two separate network branches, one with a pixel-level and one with a segment-wise loss. The same authors introduced a similar segment-level approach in^33^, whereas Mou et al.^34^ employed a multi-scale Dice loss. Zhao et al.^35^ proposed a combination of global pixel-level loss and local matting loss. Zhang and Chung^36^ introduced a deeply supervised approach in which various loss values extracted at different stages of a CNN are combined and backpropagated, with artificial labels in vessel borders turning the problem into a muti-class segmentation task. Generative Adversarial Networks (GAN) have also been proposed for retinal vessel segmentation^37–40^, although without achieving widespread popularity due to inherent difficulties in training these architectures.

It is also worth reviewing efficient approaches to retinal vessel segmentation, as our contribution introduces high-performance lightweight models. These methods typically appear in works focused on retinal vessel segmentations for embedded/mobile devices. In this context, conventional unsupervised approaches are still predominant. Arguello et al.^41^ employ image filtering coupled with contour tracing. Bibiloni et al.^42^ apply simple hysteresis thresholding, whereas Xu et al.^43^ adapt Gabor filters and morphological operations for vessel segmentation in mobile devices^43^. Only recently, Laibacher et al.^44^ explored efficient CNN architectures specifically designed for vessel segmentation on eye fundus images. Their proposed M2U-Net architecture leverages an ImageNet-pretrained MobileNet model^45^ and achieves results only slightly inferior to the state-of-the-art.

### Goals and contributions

The goal of this paper is to show that (1) there is no need of designing complex CNN architectures to outperform *most current techniques* on the task of retinal vessel segmentation, and (2) when a state-of-the-art model is trained on a particular dataset and tested on images from different data sources, it can result in poor performance. On our way to establish these two facts, we make several contributions:

1. We collate the performance of 20 recent techniques published on relevant venues for vessel segmentation on three show well-established datasets, and then show that a simple cascaded extension of the U-Net architecture, referred to here as W-Net, results in outstanding performance when compared to baselines.
2. We establish a rigorous evaluation protocol, aiming to correct previous pitfalls in the area.
3. We test our approach in a large collection of retinal datasets, consisting of 10 different databases showing a wide range of characteristics, as illustrated in Fig. 1.
4. Our cross-dataset experiments reveal that domain shift can induce performance degradation in this problem. We propose a simple strategy to address this challenge, which is shown to recover part of the lost performance.
5. Finally, we also apply our technique to the related problems of Artery/Vein segmentation from retinal fundus images and vessel segmentation from OCTA imaging, matching the performance of previous approaches with models that contain much fewer parameters.

We believe that our results open the door to more systematic studies of new domain adaptation techniques in the area of retinal image analysis: because training one of our models to reach superior performance takes approximately 20 min in a single consumer GPU, our work can serve as a first step for quick design and experimentation with improved approaches that can eventually bridge the generalization gap across different data sources revealed by our experiments. To seed research in this direction, we release the code and data to reproduce our results at https://github.com/agaldran/lwnet.

## Methodology

### Baseline U-Net: structure and complexity

One of the main goals of this work is to explore the lower limits of model complexity for the task of retinal vessel segmentation. Accordingly, we consider one of the simplest and most popular architectures in the field of medical image segmentation, namely the U-Net^32^. A standard U-Net is a convolutional autoencoder built of a downsampling CNN that progressively applies a set of filters to the input data while reducing its spatial resolution, followed by an upsampling path that recovers the original size. U-Nets typically contain skip connections that link activation volumes from the downsampling path to the upsampling path via concatenation or addition to recover higher resolution information and facilitate gradient flow during training.

**Figure 2:**
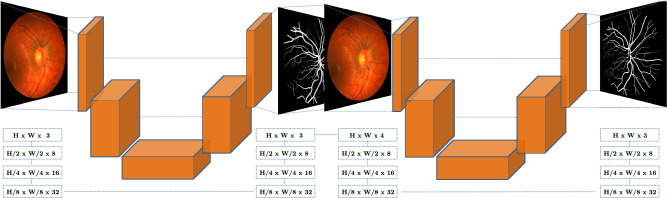
Representation of the WNet architecture. The left-hand-side part of the architecture corresponds to a standard minimal U-Net $\phi _{3,8}$ with $\sim$ 34 K parameters, which achieves performance on-par with the state-of-the-art. The full W-Net, defined by Eq. (1), is composed of two consecutive U-Nets; it outperforms all previous approaches with just around 70 k parameters: 1–3 orders of magnitude less than previously proposed CNNs.

Let us parametrize a U-Net architecture $\phi$ by the number of times the resolution is downscaled/upscaled *k*, and the number of filters applied in each of these depth levels, $f_k$. To simplify analysis, we only consider filters of size $3\times 3$, and we double the amount of filters each time we increase *k*—a common pattern in U-Net designs. Therefore, in this work a U-Net is fully specified by a pair of numbers (*k*, $f_0$), and we denote it by $\phi _{k,f_0}$. In addition, we assume that Batch-Norm layers are inserted after each convolutional operation and that extra skip connections are added within each block. An example of such design pattern is shown in the left hand side of Fig. 2. In this work, we consider the $\phi _{3,8}$ architecture, which contains approximately 34,000 parameters. It is important to stress that this represents 1–3 orders of magnitude less parameters than previously proposed CNNs for the task of retinal vessel segmentation.

### The W-Net architecture

To reach higher levels of accuracy without sacrificing simplicity, we make use of a straightforward modification of the U-Net architecture, that we refer to as W-Net. W-Net-like cascaded architectures are simple stacked U-Nets that have been widely explored in the past^46^, the motivation being that the final prediction of a model might benefit from knowing model beliefs on the value of nearby labels. The W-Net architecture is a particular case of stacked U-Nets with only two sub-networks. In this situation, the idea behind a W-Net, denoted by $\Phi$, becomes straightforward: for an input image *x*, the result of forward-passing it through a standard U-Net $\phi ^1(x)$ is concatenated to *x*, and passed again through a second U-Net, which would be represented as:

1 $$\begin{aligned} \Phi (x) = \phi ^2(x, \phi ^1(x)) \end{aligned}$$

In practice, $\phi ^1$ generates a first prediction of vessels localization that can then be used by $\phi ^2$ as a sort of attention map to focus more on interesting areas of the image, as shown in Fig. 2. Of course a W-Net $\Phi$ contains twice the amount of learnable parameters as a standard U-Net. However, since the base U-Nets $\phi _{3,8}^1, \phi ^2_{3,8}$ involved in its definition contain only 34,000 each, the W-Net considered in this paper will have around 68,000 weights, which is still one order of magnitude below the simplest architecture proposed to date for vessel segmentation, and three orders of magnitude smaller than state-of-the-art architectures.

**Figure 3:**
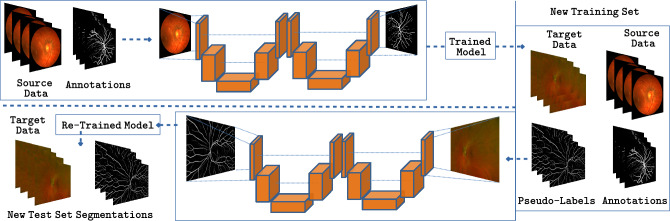
Domain Adaptation strategy employed in this work: a model trained on source data is used to generate Pseudo-Labels on a target dataset. The original source data and the target data with Pseudo-Labels are used to fine-tune the model and produce better predictions.

### Training protocol

In all the experiments reported in this paper, the training strategy remains the same. Specifically, we minimize a standard cross-entropy loss between the predictions of the model on an image *x* and the actual vessel annotations *y* (label). It is worth mentioning that in the W-Net case, an auxiliary loss is computed for the output of the first network and linearly combined with the loss computed for the second network:

2 $$\begin{aligned} \mathcal {L}(\Phi (x), y) = \mathcal {L}(\phi ^1(x), y) + \mathcal {L}(\phi ^2(x), y) \end{aligned}$$

The loss is back-propagated and minimized by means of the Adam optimization technique. The learning rate is initially set to $\lambda =10^{-2}$, and cyclically annealed following a cosine law until it reaches $\lambda =10^{-8}$. Each cycle runs for 50 epochs, and we adjust the amount of cycles (based on the size of each training set) so that we reach 4000 iterations in every experiment.

Images are all resized to a common resolution and processed with standard data augmentation techniques, and the batch size is set to 4 in all experiments. During training, at the end of each cycle, the Area Under the ROC curve is computed on a separate validation set, and the best performing model is kept. Test-Time-Augmentations (horizontal and vertical image flips) are applied during inference in all our experiments.

### A simple baseline for domain adaptation

One of the main goals in this paper is to show that, even if simple approaches can outperform much more complex current techniques, the problem of retinal vessel segmentation is not as trivial as we may extrapolate from this. The reason is that models trained on a given dataset do not reach the same level of performance when tested on retinal images sampled from markedly different distributions, as we quantitatively show later in our experiments. A relevant drop of performance appears when a model trained on a given source dataset $\mathcal {S}$ is used to generate segmentations on a substantially different target dataset $\mathcal {T}$.

Attempting to close such performance gap is a task falling within the area of Domain Adaptation, which has been subject of intensive research in the computer vision community for the last years^47^. Here we explore a simple solution to address this challenge in the context of retinal vessel segmentation. Namely, given a model $U_\mathcal {S}$ trained on $\mathcal {S}$ we proceed by first generating probabilistic segmentations for each image $x\in \mathcal {T}$. We then merge the source dataset labels $y_\mathcal {S}$ with the target dataset segmentations $\{U_\mathcal {S}(x) \ | \ x \in \mathcal {T}\}$, which we treat as Pseudo-Labels. Lastly, we fine-tune $U_\mathcal {S}$ in this new dataset, starting from the weights of the model trained on $\mathcal {S}$, with a learning rate reduced by a factor of 100, for 10 extra epochs. During training, we monitor the AUC computed in the training set (including both source labels and target Pseudo-Labels) as a criterion for selecting the best model. It is worth stressing that Pseudo-Labels $U_\mathcal {S}(x)$ are not thresholded, with the goal of informing the model about the uncertainty present on them. The rationale behind this is to force the new model to learn from segmentations in $\mathcal {S}$ with confident annotations, while at the same time exposing it to images from $\mathcal {T}$ before testing. A graphical overview of this strategy is shown in Fig. 3.

### Evaluation protocol

Unfortunately, a rigorous evaluation protocol for retinal vessel segmentation is missing in the literature due to several issues: differences in train/test splits in common benchmarks, or wrongly computed performance metrics. Below we outline what we understand as a strict evaluation protocol:

1. All performance metrics are computed at native image resolution and excluding pixels outside the Field of View, which can be trivially predicted as having zero probability of being part of a vessel.
2. Whenever an official train/test split exists, we follow it. When there is none, we follow the least “favorable” split we could find in literature: the one assigning less images for training. We make this decision based on the low difficulty of the vessel segmentation task; this is in contrast with other works that employ leave-one-out cross-validation, which can use up to $95\%$ of the data for training^31,48^.
3. We first accumulate all probabilities and labels across the training set, then perform AUC analysis and derive an optimal threshold (maximizing the Dice score) to binarize predictions. We then apply the same procedure on the test set, now using the pre-computed threshold to binarize test segmentations. This stands opposed to computing metrics per-image and reporting the mean performance^49^, or using a different threshold on each test image for binarizing probabilistic predictions^50^.
4. Cross-dataset experiments are reported in a variety of different datasets. No pre-processing or hyper-parameters are re-adjusted when changing datasets, since this heavily undermines the utility of a method. This is a typical shortcoming of unsupervised approaches, which tend to modify certain parameters to account for different vessel calibers^6^. Also, the threshold to binarize predictions on different datasets is the one derived from the original training set, without using test data to readjust it.
5. We do not report accuracy, since this is a highly imbalanced problem; the Dice score is a more suitable figure of merit. We also report Matthews Correlation Coefficient (MCC), as it is better suited for imbalanced problems^51^. Sensitivity and specificity computed at a particular cut-off value are avoided, as they are less useful when comparing the performance of different models.

## Experimental results

In this section, we provide a comprehensive performance analysis of the methodology introduced above.

### Datasets

A key aspect of this work is our performance analysis on a wide range of data sources. For intra-database tests, we compare existing results in literature with each proposed model in this work, using three different public datasets: DRIVE^1^, CHASE-DB^2^ and HRF^3^. The train/validation/test splits for DRIVE are provided by the authors. We adopt the most restrictive splits we could find in the literature for the other two datasets^44^: only 8 of the 22 images in CHASE-DB, and 15 of the 45 images in HRF are used for training and validation. After training, we test our models on the corresponding (intra-database) test sets.

In our domain adaptation experiments, we also consider seven different datasets to evaluate cross-database and the proposed technique. These include a variety of different image qualities, resolutions, pathologies, and image modalities. Further details of each of these databases are given in Table 1.

**Table 1:** Description of each of the ten datasets considered in this paper in terms of image and population characteristics.

	Year	# ims.	Resolution	FOV	Challenges & Comments
STARE^4^	2000	20	605 $\times$ 700	35$^{\circ }$	Poor quality: scanned and digitized photographs Healthy and pathological images (10/10)
DRIVE^1^	2004	40	565 $\times$ 584	45$^{\circ }$	Consistent good quality and contrast, low resolution Mostly healthy patients, some with mild DR (33/40)
CHASE-DB 1^2^	2012	28	999 $\times$ 960	30$^{\circ }$	OD-centered images from 10-year old children Uneven background illumination and poor contrast
HRF^3^	2013	45	3504 $\times$ 2336	60$^{\circ }$	High visual quality, images taken with mydriatic dilation Healthy, diabetic, and glaucomatous patients (15/15/15)
DRiDB^9^	2013	50	720 $\times$ 576	45$^{\circ }$	Highly varying quality, illumination, and image noise Mostly diabetic patients of varying grades (36/50)
AV-WIDE^8^	2015	30	2816 $\times$ 1880 1500 $\times$ 900	200$^{\circ }$	Uneven illumination, varying resolution due to cropping Healthy and age-related macular degeneration patients.
IOSTAR^6^	2016	30	1024 $\times$ 1024	45$^{\circ }$	Scanning Laser Ophthalmoscope images Macula-centered, high contrast and visual quality
DR HAGIS^7^	2017	40	2816 $\times$ 1880 4752 $\times$ 3168	45$^{\circ }$	Multi-center, multi-device macula-centered images All diabetic patients with different co-morbities
UoA-DR^10^	2017	200	2124 $\times$ 2056	45$^{\circ }$	Both macula and OD-centered images Healthy, NP-DR and P-DR patients (56/114/30)
LES-AV^5^	2018	22	1144 $\times$ 1620 1958 $\times$ 2196	30$^{\circ }$ 45$^{\circ }$	OD-centered images, highly varying illumination 11 healthy and 11 glaucomatous patients

It is worth mentioning that, for training, all images from DRIVE, CHASEDB, and HRF are downsampled to a $512\times 512$, $512\times 512$, and $1024\times 1024$ resolution respectively, whereas evaluation is carried out at native resolution for all datasets. No pre-processing (nor post-processing) was applied.

### Performance evaluation

For evaluating our approach, we follow the procedure outlined in the previous section, and report AUC, Dice, and MCC values in Table 2. For comparison purposes, we select a large set of 20 vessel segmentation techniques published in the last years in relevant venues. We also report the performance of a standard U-Net $\phi _{3,8}$, which contains around 34,000 parameters, and the proposed W-Net (with twice as many parameters), referred to as Little U-Net/W-Net respectively. In addition, for the Little W-Net case, we run the experiments five times with a different random seed, collect the results, and report the average performance. We also show a $95\%$ confidence interval for those performances in Table 3, which contains the true average with a probability of $p = 0.95$, under the assumption of normally distributed scores. In Table  2, underlined performances lie within the confidence intervals of the Little W-Net corresponding performance.

**Table 2:** Performance comparison of methods trained/tested on DRIVE, CHASE-DB, and HRF.

	# Pub/Year	# Params	DRIVE			CHASE-DB			HRF		
			AUC	Dice	MCC	AUC	Dice	MCC	AUC	DICE	MCC
Maninis et al.^24^	**ECCV/2016**	–	–	82.20	–	–	–	–	–	–	–
Zhang et al.^6^	**TMI/2016**	–	96.36	–	–	96.06	–	–	96.08	–	74.10
Fu et al.^25^	**MICCAI/2016**	–	94.04	78.75	–	94.82	75.49	–	–	–	–
Liskowski et al.^23^	**TMI/2016**	48,000,000	97.90	–	–	98.45	–	–	–	–	–
Orlando et al.^22^	**TBME/2017**	–	95.07	78.57	75.56	95.24	73.32	70.46	95.24	71.58	68.97
Gu et al.^52^	**TMI/2017**	–	–	78.86	75.89	–	72.02	69.08	–	77.49	75.41
Wu et al.^53^	**MICCAI/2018**	–	98.07	–	–	98.25	–	–	–	–	–
Yan et al.^31^	**TBME/2018**	–	97.52	81.83	–	97.81	–	–	–	78.14	–
Wang et al.^54^	**BSPC/2019**	–	–	81.44	78.95	–	78.63	76.55	–	–	–
Wang et al.^55^	**MICCAI/2019**	–	97.72	82.70	–	98.12	80.37	–	–	–	–
Araujo et al.^56^	**MICCAI/2019**	–	97.90	–	–	98.20	–	–	–	–	–
Fu et al.^57^	**MICCAI/2019**	–	97.19	80.48	–	–	–	–	–	–	–
Wang et al.^58^	**PatRec/2019**	–	–	80.93	78.51	–	78.09	75.91	–	77.31	–
Wu et al.^59^	**TMI/2019**	–	97.79	–	–	–	–	–	–	–	–
Zhao et al.^39^	**TMI/2019**	–	–	78.82	–	–	–	–	–	76.59	–
Laibacher et al.^44^	**CVPR-W/2019**	549,748	97.14	80.91	–	97.03	80.06	–	–	78.14	–
Shin et al.^26^	**MedIA/2019**	7,910,000	98.01	82.63	–	98.30	80.34	–	**98.38**	**81.51**	–
Zhao et al.^35^	**PatRec/2020**	–	–	82.29	–	–	–	–	–	77.31	–
Zhuo et al.^50^	**CMPB/2020**	–	97.54	81.63	–	–	–	–	–	–	–
Mou et al.^34^	**TMI/2020**	56,030,000	97.96	–	–	98.12	–	–	–	–	–
**Little U-Net**		34,201	97.98	82.41	79.81	98.22	80.29	78.23	98.11	80.59	78.60
**Little W-Net**		68,482	**98.10**	**82.79**	**80.24**	**98.47**	**81.69**	**79.74**	98.25	81.03	**79.09**

Best results are marked bold. A result is underlined whenever it lies within the confidence interval of the Little W-Net model (specified in Table 3 below).

**Table 3:** Average performance of the Little W-Net model for each of the datasets in Table 2 over 5 training runs, with a confidence interval containing the true mean with probability $p = 95\%$, under a normality assumption of the performances.

DRIVE			CHASE-DB			HRF		
AUC	DICE	MCC	AUC	DICE	MCC	AUC	DICE	MCC
$98.10\pm 0.04$	$82.79\pm 0.11$	$80.24\pm 0.12$	$98.47\pm 0.02$	$81.69\pm 0.22$	$79.74\pm 0.23$	$98.25\pm 0.05$	$81.03\pm 0.08$	$79.09\pm 0.09$

As discussed above, not all techniques were trained on the same data splits for CHASE-DB and HRF datasets. Our splits correspond to those used in^44^, which is a model specifically designed to be efficient, and therefore contains a minimal amount of learnable parameters. Surprisingly, we see that the Little U-Net model surpasses the performance of^44^ in all datasets, even if it has $\sim 16$ times less weights. We note results for Little U-Net are already remarkable at this stage, achieving a performance on-par or superior to most of the compared techniques.

When we analyze the performance of the Little W-Net model, we observe that it surpasses, by a wide margin, both in terms of AUC and Dice score, the numbers obtained by all the other techniques. This is specially remarkable when considering that the Little W-Net is a far less complex model than any other approach (excluding Little U-Net). The only dataset where Little W-Net fails to reach the highest performance is HRF, which we attribute to the mismatch in training and test resolutions. The work in^26^, which achieves the state-of-the-art in this dataset, was trained on image patches, and it is therefore less susceptible to such mismatch. Nevertheless, the Little W-Net achieves the second best ranking in this dataset, within a short distance from^26^.

### Cross-dataset experiments and domain adapation

From the above analysis, one could be tempted to conclude that the task of segmenting the vasculature on retinal images is practically solved. Nevertheless, the usefulness of these models remains questionable if they are not tested for their generalization capabilities beyond intra-dataset benchmarks. To exhaustively explore this aspect, we select the W-net model trained on DRIVE images, and generate predictions on up to ten different datasets (including the DRIVE test set). We then carry out a performance analysis on each external test set similar to the one described above, and report the results in the first row of Table 4. One can observe how the apparently remarkable performance of this model on the intra-DRIVE test is only maintained on the STARE dataset, which is quite similar in terms of resolution and quality. As a general rule, the performance degrades in a cross-database generalization test. In terms of AUC, the four worst results correspond to: (1) HRF, which has images of a much greater resolution than DRIVE, (2) LES-AV, where images are centered in the optic disc instead of in the macula, (3) AV-WIDE, which contains ultra-wield field images of markedly different aspect, and 4) UoA-DR, which has mostly pathological images of different resolutions.

**Table 4:** Our domain adaptation strategy improves results in a wide range of external test sets.

Training set	DRIVE			CHASE-DB			HRF			STARE			IOSTAR		
	AUC	DICE	MCC	AUC	DICE	MCC	AUC	DICE	MCC	AUC	DICE	MCC	AUC	DICE	MCC
DRIVE	**98.09**	**82.82**	**80.27**	97.22	75.13	72.44	95.90	70.39	68.05	98.11	79.48	77.30	97.97	78.77	76.47
PSEUDO-L	**98.09**	**82.82**	**80.27**	**97.56**	**76.49**	**74.02**	**96.12**	**71.12**	**68.86**	**98.28**	**79.76**	**77.65**	**98.06**	**78.95**	**76.73**

Training Set	DRiDB			LES-AV			DR HAGIS			AV-WIDE			UoA-DR		
	AUC	DICE	MCC	AUC	DICE	MCC	AUC	DICE	MCC	AUC	DICE	MCC	AUC	DICE	MCC
DRIVE	96.17	**68.45**	**66.62**	95.45	76.60	74.32	97.17	67.92	66.79	86.54	61.51	59.02	82.32	**38.29**	**35.51**
PSEUDO-L	**96.52**	68.25	66.59	**97.34**	**77.93**	**75.92**	**97.34**	**68.67**	**67.49**	**87.64**	**62.46**	**59.97**	**82.71**	37.68	34.97

First row: W-Net trained on DRIVE, second row (Pseudo-Labels): same model fine-tuned using the strategy illustrated in Fig. 3. Best metric marked in bold. Please note that Dice/MCC are computed in all cases from segmentations binarized using a threshold that is optimal for maximizing the Dice score in the training dataset (DRIVE).

**Figure 4:**
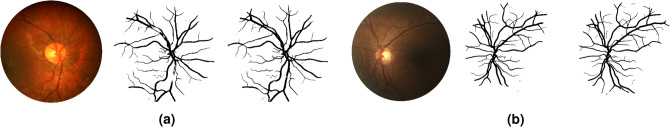
The proposed Domain Adaptation strategy recovers some missing vessels. Segmentations produced by a model trained on DRIVE (which contains macula-centered images) when using data from CHASE-DB and LES-AV (which contain OD-centered images). In (**a**,**b**), the retinal image (left), the segmentation by the model trained on DRIVE (center) and the one produced by the model trained on pseudo-labels (right).

We then apply the strategy described in the previous Section: for each dataset we use the model trained on DRIVE to generate segmentations that we use as Pseudo-Labels to retrain the same model in an attempt to close the performance gap. Results of this series of experiments are displayed in the second row of Table 4, where it can be seen that in almost all cases this results in an increased performance in terms of AUC, Dice score, and MCC, albeit relatively modest in some datasets. In any case, this implies that the retrained models have a better ability to predict vessel locations on new data. Figure 4 illustrates this for two images sampled from the CHASE-DB and the LES-AV datasets. Note that DRIVE does not contain optic-disc centered images. For the CHASE-DB example, we see that some broken vessels, probably due to the strong central reflex in this image, are recovered with the adapted model. In the LES-AV case, we see how an image with an uneven illumination field results in the DRIVE model missing much of the vessel pixels in the bottom area. Again, part of this vessels are successfully recovered by the adapted model.

### Artery/vein segmentation

We also provide results for the related problem of Artery/Vein segmentation. It should be stressed that this is a different task than A/V classification, where the the vessel tree is assumed to be available, and the goal is to classify each vessel pixel among the two classes. In this case, we aim to classify each pixel in the entire image as artery, vein, or background. In order to account for the increased complexity, we consider a bigger W-Net composed of two U-Nets $\phi _{4,8}$, which still contains far less weights than current A/V segmentation models^60,61^. In addition, we double the number of training cycles, and train with 4 classes having into account uncertain pixels, as it has been proven beneficial for this task^60^.

Table 5 shows the results of the proposed W-Net, compared with two recent A/V segmentation techniques. In this section, we train our model on DRIVE and HRF, following the data splits provided in^61^. We also show results of a cross-dataset experiment in which a model trained on DRIVE is tested on the LES-AV dataset.

**Table 5:** Performance comparison for the artery/vein segmentation task.

	# Params	DRIVE		HRF		LES-AV$^*$	
		DICE	MCC	DICE	MCC	DICE	MCC
Galdran et al.^60^	$\sim$29M	96.31 | **96.25**	74.79 | 25.07	–	–	**96.59**	**70.58**
Hhemelings et al.^61^	$\sim$5M	**96.71** | 95.81	77.57 | 24.67	96.88	**76.89**	–	–
**W-Net**	$\sim$279K	96.69 | 95.55	**77.73** | **25.23**	**96.89**	76.19	96.46	70.30

For DRIVE, performance is reported on the entire image domain | on a ring-shaped region around the Optic Disc^61^. Performance is computed using the predictions and code provided by^61^. $^*$Predictions on LES-AV are generated from models trained on DRIVE.

**Figure 5:**
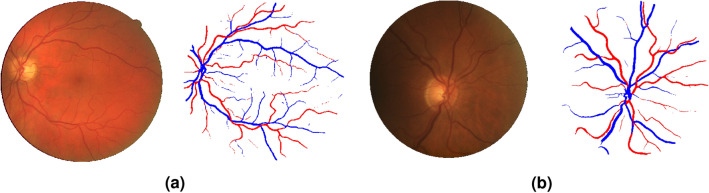
Generalization ability of a W-Net trained for A/V segmentation. Results of our model trained on DRIVE and tested on (**a**) DRIVE, (**b**) LES-AV.

A similar trend as in the previous section can be observed also here: other models designed for the same task contain orders of magnitude more parameters than the proposed approach, but we observe an excellent performance of the W-Net architecture: it seems competitive with the compared methods, ranking even higher than^61^ in terms of Dice score, and higher than^60^ in terms of MCC, at a fraction of computational cost. Some qualitative results of the W-Net trained on DRIVE and tested on LES-AV are shown in Fig. 5.

### OCTA segmentation

**Figure 6:**
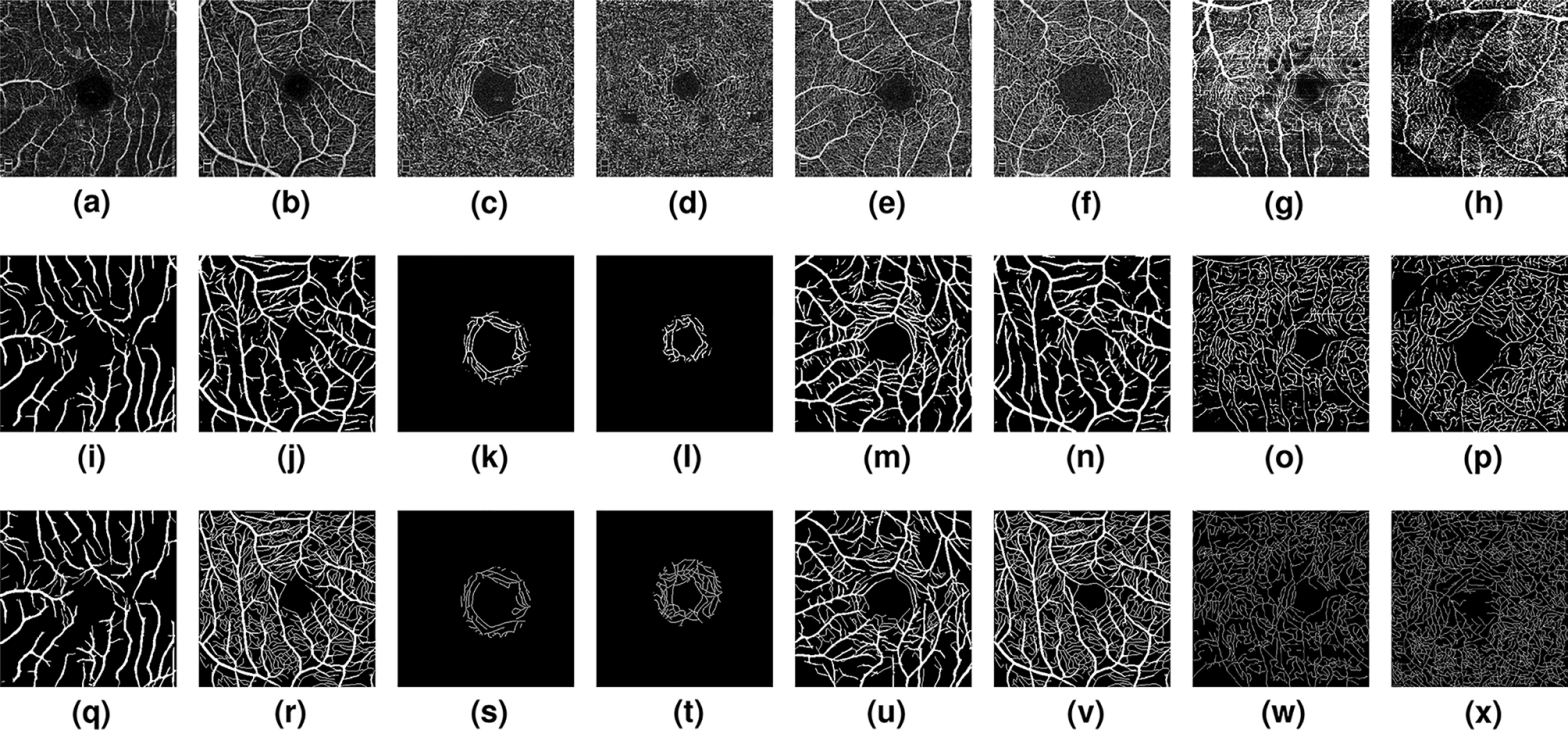
OCTA vessel segmentation. (**a**,**b**): SVC images, (**c**,**d**): DVC images, (**e**,**f**): SVC+DVC images, (**h**,**i**): Rose-2 images. The second row shows predicted probabilities and the third rows corresponding manual ground-truths. Each pair shows representative best and worst case segmentations in the corresponding test set.

While color fundus images represent the most common retinal imaging modality, there are better alternatives when the goal is to capture thin vasculature from the foveal area. Increasingly, Optical Coherence Tomography Angiography (OCTA) has been emerging as an ideal imaging technique that can generate high-resolution visualizations of the retinal vasculature. OCTA images are natively 3D, but 2D *en face* images are typically obtained from the acquired volumes by vertically projecting different OCTA flow signals within specific slices, which allows to visualize Superficial Vascular Complexes (SVC), Deep Vascular Complexes (DVC), or the inner retinal vascular plexus, which comprises both SVC and DVC (SVC+DVC). However, OCTA images (and their 2D projections) are often noisy and hard to process, as can be appreciated in the top row of Fig. 6. Therefore, they represent an ideal test scenario to measure the efficacy of the proposed minimalistic network for segmenting vessel-like structures.

In this case, we rely on a recently released database of OCTA images, the ROSE dataset^62^. ROSE is composed of two sub-datasets: ROSE-1 contains 117 OCTA images from 39 subjects, acquired with an RTVue XR Avanti SD-OCT system (Optovue, USA), at an image resolution of $304\times 304$, with SVC, DVC and SVC+DVC angiograms. ROSE-2 contains 112 OCTA *en face* angiograms of the SVC, reconstructed from $512\times 512$ scans, acquired with a Heidelberg OCT2 system (Heidelberg Engineering, Heidelberg, Germany). Visual characteristics of both data sources are noticeably different, as shown in Fig. 6.

For a fair comparison, in both cases we follow the same train/test splits and report the same evaluation metrics as in^62^. Performance of some competing methods are also extracted from^62^, where the interested reader can find more details about the different techniques. Performance comparison with respect to a Little W-Net model is shown in Table 6 for the SVC and SVC+DVC sections of ROSE-1, and in Table 7 for the DVC section of ROSE-1 and for ROSE-2. Note that our architecture was trained in exactly the same fashion as in the fundus images case.

**Table 6:** Performance comparison for OCTA vessel segmentation on ROSE-1 (SVC and SVC+DVC).

	# Pub/Year	ROSE-1 (SVC)						ROSE-1 (SVC+DVC)					
		AUC	DICE	ACC	G-mean	Kappa	FDR	AUC	DICE	ACC	G-mean	Kappa	FDR
IPAC^63^	**TMI/2015**	84.20	57.51	82.45	75.17	46.64	48.16	79.41	52.23	80.07	70.54	39.82	52.11
COSFIRE^18^	**MedIA/2015**	92.86	75.17	92.27	78.83	70.89	**4.71**	88.00	66.71	89.81	72.56	61.25	**9.88**
CE-Net^59^	**TMI/2019**	92.92	75.11	91.21	82.56	69.78	19.95	91.55	73.00	89.90	82.03	66.78	24.79
CS-Net^64^	**MICCAI/2019**	93.92	76.08	91.52	83.04	70.93	18.83	93.11	74.88	90.73	82.63	69.19	21.37
COOF^65^	**TMI/2020**	86.89	66.06	85.30	81.61	56.84	41.21	82.17	56.85	77.62	77.42	43.06	54.65
OCTA-Net^62^	**TMI/2020**	94.53	76.97	91.82	83.61	72.01	17.75	93.75	75.76	90.99	83.38	70.22	20.49
**Little W-Net**		**94.95**	**79.03**	**92.28**	**85.78**	**74.30**	18.20	**94.05**	**78.47**	**92.03**	**85.20**	**73.59**	18.25

Best results are marked in bold.

**Table 7:** Performance comparison for OCTA vessel segmentation on ROSE-1 (DVC) and ROSE-2.

	# Pub/Year	ROSE-1 (DVC)						ROSE-2					
		AUC	DICE	ACC	G-mean	Kappa	FDR	AUC	DICE	ACC	G-mean	Kappa	FDR
IPAC^63^	**TMI/2015**	75.63	9.11	75.22	76.84	6.36	95.10	73.70	55.15	85.92	82.07	47.58	55.90
COSFIRE^18^	**MedIA/2015**	85.20	24.05	91.30	85.23	21.99	85.16	77.87	61.42	92.12	77.42	56.99	38.91
CE-Net^59^	**TMI/2019**	95.05	57.83	98.43	85.03	57.07	51.47	84.67	70.66	93.77	82.48	67.08	29.30
CS-Net^64^	**MICCAI/2019**	96.71	58,84	98.82	81.55	58.25	47.10	85.42	70.10	93.85	82.35	66.58	30.25
COOF^65^	**TMI/2020**	81.62	10.03	66.78	78.47	6.49	94.65	74.42	61.12	89.45	81.17	54.98	46.20
OCTA-Net^62^	**TMI/2020**	96.73	70.74	99.09	**88.11**	70.28	34.92	86.03	**70.77**	93.86	**83.15**	**67.24**	30.19
**Little W-Net**		**97.71**	**70.82**	**99.27**	83.87	**70.47**	**26.14**	**86.25**	69.70	**94.23**	80.78	66.46	**27.23**

Best results are marked in bold.

A detailed analysis of the numerical results displayed in Tables 6 and 7 shows a similar trend as in previous sections, namely, a minimalistic but properly trained simple architecture such as Little W-Net is enough to match and often surpass the state-of-the-art also in this problem. Specifically, results from Table 6 demonstrate that Little W-Net can outperform all other competing approaches, including OCTA-Net—a recently introduced architecture that is purposefully designed to handle OCTA imaging—in all considered metrics unless in False Discovery Rate (FDR), where the most performing method is a simple unsupervised filtering approach (COSFIRE), which may actually indicate that FDR is not a suitable metric for this task. Results on Table 7 are slightly weaker for Little W-Net, although they tend to confirm its competitive performance. It is worth noting that performance in ROSE-2 seems to degrade to a small extent, probably related to the lower visual quality of these images, as shown in Fig. 6g,h.

The remainder of this section offers an ablation study with a statistical analysis on the advantages of the W-Net architecture with respect to its single U-Net counterpart for the vessel segmentation task, and also a more detailed analysis on the computational and memory requirements of our technique.

### Ablation study: W-Net vs U-Net

As shown above, the iterative structure of the W-Net architecture helps in achieving a better performance when compared to the standard U-Net. However, it should be noted that W-Net contains twice as many weights as the considered Little U-Net. Since these are two relatively small models, it might be that U-Net is simply underfitting, and all the benefits observed in Table 2 just come from doubling the parameters and not from any algorithmic improvement.

In view of this, it is worth investigating the question of whether W-Net brings a significant improvement over a standard U-Net architecture. For this, we consider a larger U-Net $\phi _{3,12}$, which actually contains more parameters than the above W-Net (76 K vs  68 K). To determine statistically significant differences in AUC and Dice between these two models, we train them under the exact same conditions as previously, and after generating the corresponding predicted segmentations on each of the three test sets, we apply the bootstrap procedure as in^66,67^. This is, each test set is randomly sampled with replacement 100 times so that each new set of sampled data contains the same number of examples as the original set, in the same proportion of vessel/background pixels. For both models, we calculate the differences in AUCs and Dice scores. Resampling 100 times results in 100 values for performance differences. P-values are defined as the fraction of values that are negative or zero, corresponding to cases in which the better model in each dataset performed worse or equally than the other model. The statistical significance level is set to 5$\%$ and, thus, performance differences are considered statistically significant if $p < 0.05$. The resulting performance differences are reported in Table 8, were we refer to the U-Net $\phi _{3,12}$ as “Big U-Net”. We see that, in all cases, the larger U-Net’s results are slightly better than the smaller U-Net in Table 2, but the performance of the W-Net is still significantly higher, even if it has approximately $10\%$ less weights.

**Table 8:** Performance comparison between a W-Net and a U-Net configured to have a comparable amount of weights.

	# Params	DRIVE		CHASE-DB		HRF	
		AUC	DICE	AUC	DICE	AUC	DICE
**“Big” U-Net**	76,213	98.00	82.53	98.29	81.09	98.15	80.73
**Little W-Net**	68,482	**98.09**	**82.78**	**98.44**	**81.52**	**98.24**	**81.05**
**W-Net vs U-Net**	$-$ ** 7731**	**+0.09** p<0.05	**+0.25** p<0.05	**+0.15** p<0.05	**+0.43** p<0.05	**+0.09** p<0.05	**+0.32** p<0.05

W-Net achieves higher performance, despite having slightly less parameters. Statistically significant results marked bold.

### Computational and memory requirements

The reduced complexity of the models proposed in this paper enhance their suitability for resource-constrained scenarios, both in terms of training them and of deploying them in, e.g., portable devices. Training a little U-Net and a little W-Net to reach the performance shown in Table 2 is feasible even without a GPU. When training on a single GPU (GeForce RTX 2080 Ti), the training time of a little U-Net on the datasets shown in Table 2 was 24 mins (DRIVE), 22 mins (CHASE-DB) and 102 mins (HRF), whereas the little W-Net took 32 mins (DRIVE), 30 mins (CHASE-DB) and 140 mins (HRF). Regarding disk memory requirements, Table 9 shows a comparison of both architectures with another two popular models in terms of performance vs. number of parameters/disk size. We see that a little U-Net, which already attains a great performance, has the lowest disk storage space (161Kb), and the top-performant W-Net takes approximately twice this space, which is still well within limits for its deployment in embedded/portable devices. It must be noted, however, that in both cases the inference time was slightly slower when compare to other efficient approaches, partly due to implementation of Test-Time Augmentation.

**Table 9:** Parameters and memory requirements vs performance for several retinal vessel segmentation models.

	# Params	Size	DRIVE		CHASEDB		HRF	
			AUC	DICE	AUC	DICE	AUC	DICE
DRIU^24^	15M	57MB	n/a	82.20	n/a	n/a	n/a	n/a
M2U-Net^44^	0.5 M	550 kb	97.14	80.91	97.03	80.06	n/a	78.14
Little U-Net	34 K	161 kb	97.98	82.41	98.22	80.68	98.11	80.59
Little W-Net	68 K	325 kb	98.09	82.82	98.44	81.55	98.24	81.04

## Discussion

The results presented in this paper might seem surprising to the reader, and are worth further discussion. With the steady apparent improvements in the literature CNN architectures for vessel segmentation, how might it be possible that a simpler approach outperforms most recently introduced methods? For example, the technique in^31^ employs a similar architecture, but at a larger scale, and with an improved loss function that handles thin vessels in a dedicated manner, yet it appears to deliver inferior performance than the Little W-Net. We believe the reason behind the success of our approach lies on the training process, that leverages modern practices like cyclical learning rates, adequate early-stopping in terms of AUC on a separate validation set, and Test-Time Augmentation which, in our opinion should be always included. It is important to stress that this paper does not claim any superiority of the Little W-Net architecture with respect to other methods. Rather, our main message is that vessel segmentation problem from retinal fundus images can be successfully solved *on standard datasets* without complex artefacts, but such approach will unlikely generalize well. New contributions should be examined critically in the future. Connected to this, we recommend the application of meticulous evaluation protocols like the one detailed previously. In particular, some of the metrics commonly reported in previous works are of uncertain interest, and should be avoided. For example, accuracy is not a good measure in such an imbalanced problem. Reporting specificity and sensitivity conveys little information for deciding when one method is superior or not to another. We believe the combination of AUC, Dice score and Matthews Correlation Coefficient represents a better approach to performance measurement in this problem. We hope that the release of all our code will favor reproducibility and rigorous benchmarking of vessel segmentation techniques in the future.

Another point worth stressing is the role that image resolution plays in vessel segmentation. The DRIVE database, the most common benchmark, has a resolution of $584\times 565$, which is considerably far from that of state-of-the-art fundus cameras, or useful to practical applications. As argued in^68^, developing new methods guided by the performance in this database diminishes the technical advantages brought to the field by more advanced imaging instruments. We believe relatively old datasets like DRIVE or STARE have been sufficiently studied, and reporting should switch to modern high-resolution databases as soon as possible. Lack of data is not a challenge anymore, as recent but less known databases (see Table 1) are largely ignored in publications in an effort to to compare with previous approaches. Our results on a large number of data sources may encourage research in this direction. In this sense, note that for the CHASE-DB and HRF databases, our architecture was trained on downsampled images, which were approximately half the native resolution of the original samples (although all tests were carried out at native resolution by a posteriori upsampling of the predictions), which is a relevant limitation. Results in this paper provide an adequate baseline from which to improve performance based, e.g., on the design of smart patch-based methods that can handle varying resolutions seamlessly, or more exotic super-resolution approaches.

The minimal size of the models introduced in this paper enables relevant applications. A Little W-Net takes up a disk space of 161Kb, which turns it into an ideal candidate for its deployment on portable devices. The the reduced number of parameters allowed us to duplicate the size of a standard U-Net, which brought noticeable performance improvements at an acceptable computational cost.

## Conclusions

This paper reflects on the need of constructing algorithmically complex methodologies for the task of retinal vessel segmentation. In a quest for squeezing an extra drop of performance on public benchmark datasets and adding certain novelty, recent approaches for this topic show a trend on developping overcomplicated pipelines that may not be necessary for this task. The first conclusion to be drawn from our work is that sometimes Occam’s razor works best: minimalistic models, properly trained, can attain results that do not significantly differ from what one can achieve with more complex approaches.

Another point worth stressing is the need of rigor in evaluating retinal vessel segmentation techniques. Employing overly favorable train/test splits or incorrectly computing performance leads to reporting inflated metrics, which in turn saturate public benchmarks and provides a false confidence that the retinal vessel segmentation is unchallenging. Our experiments on a wide range of datasets reveal that this is not the case, and that retinal vessel segmentation is indeed an ideal area for experimenting with domain adaptation techniques. This is so because a) performance of models trained on a source dataset rapidly degrades when testing on a different kind of data, and b) training models to achieve high performance is cheap and fast, which enables fast experimentation of new ideas.

## References

[1] Staal J, Abramoff M, Niemeijer M, Viergever M, van Ginneken B (2004). Ridge-based vessel segmentation in color images of the retina. IEEE Trans. Med. Imaging.

[2] Fraz MM (2012). An ensemble classification-based approach applied to retinal blood vessel segmentation. IEEE Trans. Biomed. Eng..

[3] Budai A, Bock R, Maier A, Hornegger J, Michelson G (2013). Robust vessel segmentation in fundus images. Int. J. Biomed. Imaging.

[4] Hoover A, Kouznetsova V, Goldbaum M (2000). Locating blood vessels in retinal images by piecewise threshold probing of a matched filter response. IEEE Trans. Med. Imaging.

[5] Orlando JI, Frangi AF, Schnabel JA, Davatzikos C, Alberola-López C, Fichtinger G (2018). Towards a glaucoma risk index based on simulated hemodynamics from fundus images. Medical Image Computing and Computer Assisted Intervention—MICCAI 2018.

[6] Zhang J (2016). Robust retinal vessel segmentation via locally adaptive derivative frames in orientation scores. IEEE Trans. Med. Imaging.

[7] Holm S, Russell G, Nourrit V, McLoughlin N (2017). DR HAGIS-a fundus image database for the automatic extraction of retinal surface vessels from diabetic patients. J. Med. Imaging (Bellingham, Wash.).

[8] Estrada R (2015). Retinal artery-vein classification via topology estimation. IEEE Trans. Med. Imaging.

[9] Prentašić, P. *et al.* Diabetic retinopathy image database(DRiDB): A new database for diabetic retinopathy screening programs research. In *2013 8th International Symposium on Image and Signal Processing and Analysis (ISPA)*, 711–716. 10.1109/ISPA.2013.6703830 (2013). ISSN: 1845-5921.

[10] Chalakkal, R. J., Abdulla, W. H. & Sinumol, S. Comparative analysis of University of Auckland Diabetic Retinopathy Database. In *Proceedings of the 9th International Conference on Signal Processing Systems*, ICSPS 2017, 235–239. 10.1145/3163080.3163087 (Association for Computing Machinery, Auckland, New Zealand, 2017).

[11] Niemeijer M (2011). Automated measurement of the arteriolar-to-venular width ratio in digital color fundus photographs. IEEE Trans. Med. Imaging.

[12] Welikala RA (2016). Automated retinal image quality assessment on the UK Biobank dataset for epidemiological studies. Comput. Biol. Med..

[13] Chen L, Huang X, Tian J (2015). Retinal image registration using topological vascular tree segmentation and bifurcation structures. Biomed. Signal Process. Control.

[14] Costa P (2018). End-to-end adversarial retinal image synthesis. IEEE Trans. Med. Imaging.

[15] Zana F, Klein J-C (2001). Segmentation of vessel-like patterns using mathematical morphology and curvature evaluation. IEEE Trans. Image Process..

[16] Mendonca A, Campilho A (2006). Segmentation of retinal blood vessels by combining the detection of centerlines and morphological reconstruction. IEEE Trans. Med. Imaging.

[17] Frangi AF, Niessen WJ, Vincken KL, Viergever MA, Wells WM, Colchester A, Delp S (1998). Multiscale vessel enhancement filtering. Medical Image Computing and Computer-Assisted Intervention—MICCAI’98.

[18] Azzopardi G, Strisciuglio N, Vento M, Petkov N (2015). Trainable COSFIRE filters for vessel delineation with application to retinal images. Med. Image Anal..

[19] Soares J, Leandro J, Cesar R, Jelinek H, Cree M (2006). Retinal vessel segmentation using the 2-D Gabor wavelet and supervised classification. IEEE Trans. Med. Imaging.

[20] Marín D, Aquino A, Gegundez-Arias ME, Bravo JM (2011). A new supervised method for blood vessel segmentation in retinal images by using gray-level and moment invariants-based features. IEEE Trans. Med. Imaging.

[21] Becker C, Rigamonti R, Lepetit V, Fua P, Mori K, Sakuma I, Sato Y, Barillot C, Navab N (2013). Supervised feature learning for curvilinear structure segmentation. Medical Image Computing and Computer-Assisted Intervention—MICCAI 2013.

[22] Orlando JI, Prokofyeva E, Blaschko MB (2017). A discriminatively trained fully connected conditional random field model for blood vessel segmentation in fundus images. IEEE Trans. Bio-med. Eng..

[23] Liskowski P, Krawiec K (2016). Segmenting retinal blood vessels with deep neural networks. IEEE Trans. Med. Imaging.

[24] Maninis K-K, Pont-Tuset J, Arbeláez P, Van Gool L, Ourselin S, Joskowicz L, Sabuncu MR, Unal G, Wells W (2016). Deep retinal image understanding. Medical Image Computing and Computer-Assisted Intervention—MICCAI 2016.

[25] Fu H, Xu Y, Lin S, Kee Wong DW, Liu J, Ourselin S, Joskowicz L, Sabuncu MR, Unal G, Wells W (2016). DeepVessel: Retinal vessel segmentation via deep learning and conditional random field. Medical Image Computing and Computer-Assisted Intervention—MICCAI 2016.

[26] Shin SY, Lee S, Yun ID, Lee KM (2019). Deep vessel segmentation by learning graphical connectivity. Med. Image Anal..

[27] Guo Y, Peng Y (2020). BSCN: Bidirectional symmetric cascade network for retinal vessel segmentation. BMC Med. Imaging.

[28] Fan, Z. *et al.* Accurate retinal vessel segmentation via octave convolution neural network. (2019). arXiv:1906.12193.

[29] Wang, K., Zhang, X., Huang, S., Wang, Q. & Chen, F. CTF-Net: Retinal vessel segmentation via deep coarse-to-fine supervision network. In *2020 IEEE 17th International Symposium on Biomedical Imaging (ISBI)*, 1237–1241. 10.1109/ISBI45749.2020.9098742 (2020). ISSN: 1945-8452.

[30] Cherukuri V, Kumar BG, Bala VR, Monga V (2020). Deep retinal image segmentation with regularization under geometric priors. IEEE Trans. Image Process..

[31] Yan Z, Yang X, Cheng K-T (2018). Joint segment-level and pixel-wise losses for deep learning based retinal vessel segmentation. IEEE Trans. Biomed. Eng..

[32] Ronneberger O, Fischer P, Brox T, Navab N, Hornegger J, Wells WM, Frangi AF (2015). U-Net: Convolutional networks for biomedical image segmentation. Medical Image Computing and Computer-Assisted Intervention—MICCAI 2015.

[33] Yan Z, Yang X, Cheng K-T (2019). A three-stage deep learning model for accurate retinal vessel segmentation. IEEE J. Biomed. Health Inform..

[34] Mou L (2020). Dense dilated network with probability regularized walk for vessel detection. IEEE Trans. Med. Imaging.

[35] Zhao H, Li H, Cheng L (2020). Improving retinal vessel segmentation with joint local loss by matting. Pattern Recogn..

[36] Zhang Y, Chung ACS, Frangi AF, Schnabel JA, Davatzikos C, Alberola-López C, Fichtinger G (2018). Deep supervision with additional labels for retinal vessel segmentation task. Medical Image Computing and Computer Assisted Intervention–MICCAI 2018.

[37] Lahiri, A., Ayush, K., Kumar Biswas, P. & Mitra, P. Generative adversarial learning for reducing manual annotation in semantic segmentation on large scale miscroscopy images: Automated vessel segmentation in retinal fundus image as test case. In *2017 IEEE Conference on Computer Vision and Pattern Recognition Workshops (CVPRW)*, 42–48. 10.1109/CVPRW.2017.110 (2017).

[38] Son J, Park SJ, Jung K-H (2019). Towards accurate segmentation of retinal vessels and the optic disc in fundoscopic images with generative adversarial networks. J. Digit. Imaging.

[39] Zhao H (2019). Supervised segmentation of un-annotated retinal fundus images by synthesis. IEEE Trans. Med. Imaging.

[40] Park K-B, Choi SH, Lee JY (2020). M-GAN: Retinal blood vessel segmentation by balancing losses through stacked deep fully convolutional networks. IEEE Access..

[41] Argüello F, Vilariño DL, Heras DB, Nieto A (2018). GPU-based segmentation of retinal blood vessels. J. Real-Time Image Proc..

[42] Bibiloni P, González-Hidalgo M, Massanet S (2019). A real-time fuzzy morphological algorithm for retinal vessel segmentation. J. Real-Time Image Proc..

[43] Xu X (2016). Smartphone-based accurate analysis of retinal vasculature towards point-of-care diagnostics. Sci. Rep..

[44] Laibacher, T., Weyde, T. & Jalali, S. M2U-Net: Effective and efficient retinal vessel segmentation for real-world applications. In *2019 IEEE/CVF Conference on Computer Vision and Pattern Recognition Workshops (CVPRW)*, 115–124. 10.1109/CVPRW.2019.00020 (2019). ISSN: 2160-7516.

[45] Sandler, M., Howard, A. G., Zhu, M., Zhmoginov, A. & Chen, L.-C. MobileNetV2: Inverted residuals and linear bottlenecks. In *2018 IEEE/CVF Conference on Computer Vision and Pattern Recognition* 4510–4520. 10.1109/CVPR.2018.00474 (2018).

[46] Havaei M (2017). Brain tumor segmentation with deep neural networks. Med. Image Anal..

[47] Kouw WM, Loog M (2019). A review of domain adaptation without target labels. IEEE Trans. Pattern Anal. Mach. Intell..

[48] Oliveira A, Pereira S, Silva CA (2018). Retinal vessel segmentation based on fully convolutional neural networks. Expert Syst. Appl..

[49] Xu X, Ding W, Abràmoff MD, Cao R (2017). An improved arteriovenous classification method for the early diagnostics of various diseases in retinal image. Comput. Methods Programs Biomed..

[50] Zhuo Z, Huang J, Lu K, Pan D, Feng S (2020). A size-invariant convolutional network with dense connectivity applied to retinal vessel segmentation measured by a unique index. Comput. Methods Programs Biomed..

[51] Chicco D, Jurman G (2020). The advantages of the Matthews correlation coefficient (MCC) over F1 score and accuracy in binary classification evaluation. BMC Genom..

[52] Gu L, Zhang X, Zhao H, Li H, Cheng L (2017). Segment 2D and 3D filaments by learning structured and contextual features. IEEE Trans. Med. Imaging.

[53] Wu Y, Xia Y, Song Y, Zhang Y, Cai W, Frangi AF, Schnabel JA, Davatzikos C, Alberola-López C, Fichtinger G (2018). Multiscale network followed network model for retinal vessel segmentation. Medical Image Computing and Computer Assisted Intervention—MICCAI 2018.

[54] Wang X, Jiang X (2019). Retinal vessel segmentation by a divide-and-conquer funnel-structured classification framework. Sig. Process..

[55] Wang B, Qiu S, He H, Shen D (2019). Dual encoding U-Net for retinal vessel segmentation. Medical Image Computing and Computer Assisted Intervention—MICCAI 2019.

[56] Araújo RJ, Cardoso JS, Oliveira HP, Shen D (2019). A deep learning design for improving topology coherence in blood vessel segmentation. Medical Image Computing and Computer Assisted Intervention–MICCAI 2019.

[57] Fu W, Breininger K, Schaffert R, Ravikumar N, Maier A, Shen D (2019). A divide-and-conquer approach towards understanding deep networks. Medical Image Computing and Computer Assisted Intervention—MICCAI 2019.

[58] Wang X, Jiang X, Ren J (2019). Blood vessel segmentation from fundus image by a cascade classification framework. Pattern Recogn..

[59] Gu Z (2019). CE-Net: Context encoder network for 2d medical image segmentation. IEEE Trans. Med. Imaging.

[60] Galdran, A., Meyer, M., Costa, P., MendonÇa & Campilho, A. Uncertainty-Aware Artery/Vein Classification on Retinal Images. In *2019 IEEE 16th International Symposium on Biomedical Imaging (ISBI 2019)*, 556–560. 10.1109/ISBI.2019.8759380 (2019). ISSN: 1945-8452.

[61] Hemelings R (2019). Artery-vein segmentation in fundus images using a fully convolutional network. Comput. Med. Imaging Graph..

[62] Ma Y (2020). ROSE: A retinal OCT-angiography vessel segmentation dataset and new model. IEEE Trans. Med. Imaging.

[63] Zhao Y, Rada L, Chen K, Harding SP, Zheng Y (2015). Automated vessel segmentation using infinite perimeter active contour model with hybrid region information with application to retinal images. IEEE Trans. Med. Imaging.

[64] Mou L, Shen D (2019). CS-Net: Channel and spatial attention network for curvilinear structure segmentation. Medical Image Computing and Computer Assisted Intervention–MICCAI 2019.

[65] Zhang J (2020). 3D shape modeling and analysis of retinal microvasculature in OCT-angiography images. IEEE Trans. Med. Imaging.

[66] Samuelson, F. & Petrick, N. Comparing image detection algorithms using resampling. In *3rd IEEE International Symposium on Biomedical Imaging: Nano to Macro, 2006.*, 1312–1315.10.1109/ISBI.2006.1625167 (2006). ISSN: 1945-8452.

[67] Bria A, Marrocco C, Tortorella F (2020). Addressing class imbalance in deep learning for small lesion detection on medical images. Comput. Biol. Med..

[68] Mookiah MRK (2021). A review of machine learning methods for retinal blood vessel segmentation and artery/vein classification. Med. Image Anal..

