# Author Correction: State-of-the-art retinal vessel segmentation with minimalistic models

**DOI:** 10.1038/s41598-023-40057-0

**Published:** 2023-08-07

**Authors:** Adrian Galdran, André Anjos, José Dolz, Hadi Chakor, Hervé Lombaert, Ismail Ben Ayed

**Affiliations:** 1 University of Bournemouth, Bournemouth, UK; 2 Idiap Research Institute, Martigny, Switzerland; 3 ETS Montréal, Montreal, Canada; 4 Diagnos Inc., Quebec, Canada

Correction to: *Scientific Reports* 10.1038/s41598-022-09675-y, published online 13 April 2022 

The Funding section in the original version of this Article was omitted. The Funding section now reads:

“Adrian Galdran is funded by the EU's Horizon 2020 R&I programme under the MSC grant agreement No 892297.”

The original Article has been corrected.

